# Society Meetings

**Published:** 1915-07

**Authors:** 


### SOCIETY MEETINGS

---

**Brief reports and announcements of meetings of societies of Western New York, and those of general scope, are requested from Secretaries. Copy should be on hand the fifteenth of the month. Full transactions will be published at cost of composition.**

---

**DOCTOR: Is your Society properly represented here? If not, please bring our standing announcement to the attention of the members.**

---

At a meeting held May 5, 1915, the **New York Psychiatrical Society,** all the members of which are specialists in nervous and mental diseases who are well informed and actively interested in the State system for the care of the insane and mentally defective, for the purpose of concisely stating the views and recommendations of the Society in regard to the provisions which, in its opinion, should be made in the State Constitution for safeguarding the care and treatment of persons suffering from mental disease and defect, adopted the following preamble and resolutions:

Whereas: Experience has shown the advisability of safeguarding and shaping by constitutional requirements the essentials of an efficient and stable State system for the care of the insane and mentally defective, and

Whereas: No system can be efficient unless it is administered by specially qualified officials; therefore be it

Resolved: That this Society recommend (1) that the State Constitution require the Legislature to provide for a State Hospital Commission, the President of which shall be a physician who is specially qualified in mental diseases and in institutional administration, and for a State Commission on Mental Deficiency, the President of which shall be a physician who is specially qualified in mental deficiency and in institutional administration; (2) that the State Hospital Commission be given power not only to visit and inspect all institutions either public or private, used for the care and treatment of persons suffering from mental diseases, as at present, but be also given fiscal control of the State institutions for the care and treatment of such persons and general supervision; (3) that the State Commission on Mental Deficiency be given equal powers to those of the State Hospital Commission in regard to the institutions for the care and treatment of persons suffering from mental deficiency.

Resolved: That this Society believes that a system of fiscal control of the State institutions for the mentally diseased and mentally defective by a Board of Control which would be independent of the specially qualified Commissioners recommended in the preceding resolutions would be inefficient and would be detrimental to the proper treatment of the patients and to the interests of the State in dealing with the important problems of mental disease and deficiency.

Resolved: That the Secretary of the Society be instructed to send a copy of these resolutions to each member of the State Constitutional Convention, and that the Committee on Mental Deficiency be authorized to represent the Society in co-operating with other organizations which are interested in the Constitutional provisions relating to the mentally diseased and defective.

---

**The Interstate Association of Anaesthetists** held its first, organization meeting at Cincinnati, in conjunction with the Ohio State Medical Society, May 4. It is not a national association and does not include N. Y. but individuals may join from any state and it is hoped that similar organizations will be formed. A two-day session was held with a good attendance.

Dr. Robert Carothers predicted that anaesthesia would be used as a means of treatment and cited a case of tuberculous peritonitis apparently cured by ether administered by the closed method.

Dr. E. I. McKesson discussed blood pressure and advanced the theory that shock might be due to anaemia of the cardiac muscle due to too low pressure, so that the back flow from the aorta was not adequate to fill the coronaries.

Dr. E. M. Saunders emphasized the need of skillful specialists in anaesthesia but complained of the poor pay.

Dr. W. H. Long took a more hopeful attitude.

Dr. Chas. K. Teter deduced from his experience that nitrous oxid does not increase intra-cranial tension if the proper amount of oxygen is administered—the tension being due to partial asphyxia.

Dr. Willis D. Gatch considered shock due to decreased abdominal pressure incident to the surgical traumatism itself or to pushing anaesthesia to the point of muscular relaxation, so that too much blood accumulates in the abdominal veins. He, therefore, advocated abdominal compression after operation and had also suggested increasing intra pulmonary pressure in cardiac dilatation.

Dr. Arthur E. Guedal advocated nitrous oxid and oxygen to produce sunrise sleep as a substitute for twilight slumber. In the discussion a case of a child born cyanotic was cited, but, the cord not having been cut, the infantile circulation was restored by administering oxygen to the mother.

Dr. R. Merrill Ricketts gave credit to Dr. George Edward Fell of Buffalo for laying the foundation of forced respiration and subtracheal anaesthesia and thereby stimulating and making possible, thoracic surgery.

Dr. Edward E. Barber, a Chicago dentist, mentioned the difficulty of maintaining a state between consciousness and full anaesthesia and spoke of the possible future of local analgesia for general surgery.

The American Journal of Surgery was adopted as the official organ, it serving also as such for the American Association of Anaesthetists. The next meeting will probably be held in Columbus at the same time as the American College of Surgeons. Officers were elected as follows: Pres. W. Hamilton Long, Louisville; V. Pres. Isabella C. Herb, Chicago; Sec.-Treas. F. H. McMeehan, Cincinnati; Executive Committee: Arthur E. Guedal, Indianapolis, W. P. Burdick, Kane, Pa., Moses Sulzer, Cincinnati, W. I. Jones, Columbus, W. N. Lynn, Knoxville, John H. Evans, Buffalo. (We are indebted for this report to Dr. Evans).

---

**The Rochester Pathological Society** held its regular meeting, June 3. Dr. J. W. McGill read a paper on Acute Osteomyelitis and its Treatment.

---

**Officers of the Alumni Association, Medical Department, University of Buffalo**:

Lesser Kauffman, President, Buffalo, N. Y.
Charles D. Aaron, '91, First Vice-President, Detroit, Mich.
Archer D. Babcock, '93, Second Vice-President, Syracuse, N. Y.
James E. King, '96, Third Vice-President, Buffalo, N. Y.
Jane W. Carroll, '91, Fourth Vice-President, Paterson, N. J.
Lee A. Whitney, '01, Fifth Vice-President, Rochester, N. Y.
Julius Richter, '04, Secretary, Buffalo, N. Y.
Frank E. Brundage, '09, Treasurer, Buffalo, N. Y.

Trustees for five years:
Benjamin F. Rogers, '79 ....................... Buffalo, N. Y.
Charles L. Preisch, '98 ....................... Lockport, N. Y.
Henry C. Buswell, 88 ......................... Buffalo, N. Y.
Grover W. Wende, '89 ....................... Buffalo, N. Y.
George F. Cott, '84 .......................... Buffalo, N. Y.

Executive Committee:
W. F. Jacobs, '08, chairman, president ex-officio, Buffalo, N. Y.
Edith R. Hatch, '06, secretary ex-officio ....... Buffalo, N. Y.
Wm. G. Bissell, '92, treasurer ex-officio ........ Buffalo, N. Y.

In order to include some important announcements, the complete report of the 40th annual meeting of the Alumni will be published in the August issue.

---

**Buffalo Academy of Medicine.** May 26, Section of Pathology: A new Conception of the Physical Basis of Life, G. H. A. Clowes, Ph. D., discussion by Dr. F. A. Lidbury and Dr. F. H. Pratt. The Western New York Section of the American Chemical Society was invited.

June 8, Annual Meeting of the Academy. The retiring president, Dr. John H. Pryor read an address and reports were made by the secretaries. The address will be published later. The following officers were elected:

President, Dr. James W. Putnam; Secretary, Dr. Herbert A. Smith; Treasurer, Dr. Lawrence Hendee; Trustee (three years) Dr. Harry R. Trick.

---

**Allegany, Genesee, Livingston & Wyoming County Medical Societies** will hold a joint meeting at Glen Iris, July 15.

---

**Medical Society of the County of Erie.** By invitation of the Arts and Science Faculty of the University of Buffalo, the regular meeting was held in Townsend Hall, Delaware Avenue and Niagara Square, June 21. "Treatment of Anemias and the Hemorrhagic Diseases" by Dr. W. L. Moss, Intermist, State Institute for the Study of Malignant Disease. The Attorney of the Society, Mr. A. L. Harrison, explained the State and National Narcotic Laws. Members of the Buffalo Retail Druggists Association were present by invitation.

---

**Rupture of Both Cardiac Auricles.** A. H. Peters presented specimens to the El Paso, Col. Medical Society (page 163, Col. Med., May 1915). The patient had been killed in an automobile accident. The pericardium was not damaged.

